# The Frankfurt ‘whisper exam’ - a case-based collaborative summative examination to assess clinical decision-making skills in hygiene, microbiology and virology– feasibility and evaluation

**DOI:** 10.1186/s12909-026-10018-y

**Published:** 2026-08-01

**Authors:** Lisa Vorbeck, Miriam Ruesseler, Jan Steinmetzer, Niko Kohmer, Volkhard A. J. Kempf, Claudia Brandt

**Affiliations:** 1 Institute for Medical Microbiology and Infection Control, Frankfurt University Medicine, University Hospital, Goethe University, Paul-Ehrlich-Str. 40, Frankfurt am Main, D-60596 Germany; 2 Medical Faculty, Goethe University, Frankfurt am Main, Germany; 3 Institute for Medical Education and Clinical Simulation, Goethe University, Frankfurt am Main, Germany; 4 Institute for Medical Virology, Frankfurt University Medicine, University Hospital, Goethe University, Frankfurt am Main, Germany

**Keywords:** Case-based Examination, Key Feature Questions, Team Examination, Competency-based Examination

## Abstract

**Background:**

Collaborative assessment formats have gained increasing attention in medical education due to their potential to foster clinical reasoning, teamwork, and to reduce examination-related stress. However, evidence for their feasibility and acceptance within summative undergraduate medical curricula remains limited.

**Objective:**

This study aimed to evaluate the feasibility and student perceptions of a case-based, collaborative summative examination (“whisper exam”) in undergraduate medical education. Specifically, we explored students’ perceptions of stress, preparation effort, teamwork, and the alignment between self-assessement and actual examination performance.

**Methods:**

A single-center, descriptive feasibility study was conducted with third-year medical students (*n* = 350) following a laboratory course in hygiene, microbiology, and virology. Students completed a digital, case-based examination using key-feature questions in pairs. Examination performance was recorded at the team level. Student perceptions were collected via a post-exam voluntary questionnaire and analyzed descriptively.

**Results:**

The overall pass rate was 93.1% (326/350 students). Survey responses were obtained from 190 students (54.3%). Most respondents reported lower perceived stress compared to individual examinations and described teamwork as constructive and enjoyable. Preparation effort was reported to be comparable to that required for individual examinations. Self-assessment of performance broadly aligned with examination outcomes, although a tendency toward underestimation was observed.

**Conclusion:**

The whisper exam was feasible to implement within a summative undergraduate curriculum and positively perceived by the majority of participating students. Findings suggest that collaborative, case-based examinations may complement existing assessment formats. Further research using comparative and multi-institutional designs is required to evaluate educational impact and validity.

**Supplementary Information:**

The online version contains supplementary material available at 10.1186/s12909-026-10018-y.

## Introduction

In modern medicine, physicians are required to collaborate, think critically, and solve problems effectively even under conditions of uncertainty. Collaborative competencies are particularly crucial in cross-disciplinary medical specialties such as microbiology, virology and infection control, as infections represent integral entities of all clinical specialties and are encountered by nearly every physician.

Medical students must develop the necessary competencies to become practical and rational thinkers upon graduation [1–3]. Case-based and problem-based learning formats are particularly effective in fostering clinical reasoning and collaborative skills, as clinical problems serve as one of the most enduring and powerful triggers for both learning and knowledge retention [4, 5]. Engaging with clinical scenarios that reflect real-world practice is highly motivating for students, as students can understand the relevance of these situations to their professional work. Moreover, working through these clinical scenarios is particularly important, as they require the very competencies that students are expected to master. It directs attention to the development of problem-solving strategies, while simultaneously promoting a willingness to discuss and analyze medical issues within a collegial context. In doing so, general teamwork skills are implicitly trained, thereby laying the foundation for collaborative, respectful, and effective interaction with colleagues from various medical specialties, as well as with members of other healthcare professions.

In addition to collaborative learning formats, collaborative assessment itself is a well-established strategy for enhancing long-term knowledge retention and the acquisition of competencies [6, 7], and has gained increasing attention across multiple academic disciplines, including sciences and medicine. A collaborative exam requires students to work together to answer questions, engage in discussion, and build consensus. This format helps clarify misconceptions and reinforce learning through peer interaction. Studies have shown that collaborative exams enhance knowledge retention [8–11] and foster social and collaborative skills [12]. Working together improves communication and encourages appreciation of diverse perspectives and peer contributions. The social nature of this format can increase motivation, as students prepare more thoroughly to contribute meaningfully [13, 14]. Additionally, collaborative exams are often perceived as less stressful while enhancing motivation and learning outcomes [11].

Initial studies on the implementation of collaborative exams in undergraduate medical training suggest similar benefits as those observed in fields such as science and engineering [10, 15]. However, research specifically addressing the incorporation of collaborative exams into the formal curriculum of medical education, beyond experimental studies, remains scarce.

The present study addresses this gap by evaluating the implementation of a collaborative, case-based summative examination (“whisper exam”) in undergraduate medical education regarding feasibility and effects. The study was guided by the following research questions:

1. How do students perceive stress, preparation effort, and teamwork in a collaborative summative examination?
2. How do students evaluate the fairness and quality of collaborative decision-making during the examination?
3. How does students’ self-assessed examination performance align with objective examination outcomes?

## Project description

### Study design

This prospective single-center, descriptive feasibility study was designed using a case-based collaborative examination (Whisper Exam) as summative assessment in undergraduate medical education in the cross-disciplinary specialty of hygiene, microbiology and virology.

### Study participants

Study participants were medical students in their third year of a six-year undergraduate program at the medical faculty of Goethe University in Frankfurt, Germany. As part of their training, all students had to participate in the laboratory course of hygiene, microbiology and virology prior to taking the Whisper exam. This course consisted of 15 units in the field of hygiene, microbiology, mycology, parasitology, and virology. The concept of the course was designed as a fusion of case-based theoretical and clinical content elements with a hands-on approach to learn practical diagnostic laboratory skills in pairs of seatmates [16]. The Whisper exam was conducted simultaneously for all participants one week after completion of the practical course.

### Study protocol, assessment structure and question format

In order to assess students’ clinical reasoning and their ability to transfer knowledge acquired in the preceding laboratory course to comparable situations, the end-of-practical-training examination was redesigned to employ case-based digital assessments by using key-feature questions (KFQ). KFQ are particularly well suited for assessing clinical reasoning because they target critical decision-making moments more effectively than conventional multiple-choice questions. Their full potential is best realized in a digital examination format that enables progressive disclosure of case information [17].

The assessment evaluated in this study was made up of four clinical case vignettes comprising a total of 20 key-feature questions, each of which consisted of five response options with only one correct answer. The learning objectives assessed in the whisper exam are presented in Table 1. These objectives addressed clinical topics in medical microbiology, virology, and infection control, all of which had been covered in the preceding laboratory course mostly in a case-based format, thereby ensuring adherence to the principles of constructive alignment. The cut score was set at 12 correct answers out of 20 (60%) according to study regulations of Goethe University Frankfurt.

**Table 1:** Learning objectives assessed in the whisper examination

Students were expected to
• understand the essential pre-analytical aspects of specimen collection and transport for infectious disease diagnostics and be able to request appropriate diagnostic procedures.
• understand the different methods of direct and indirect pathogen detection using microscopic, culture-based, molecular biological, and serological techniques, including the direct agglutination test for the detection of heterophile antibodies (Paul–Bunnell test) and interferon-gamma release assays for the detection of infection with the *Mycobacterium tuberculosis*-complex.
• understand the definitions and classification of the various multidrug-resistant organisms (based on antibiotic susceptibility testing), be familiar with last-resort antibiotics, and derive appropriate infection prevention and hospital hygiene measures.
• understand the major risk factors for nosocomial infections and the measures required for the prevention and containment of healthcare-associated infectious outbreaks, including relevant mandatory reporting to public health authorities.
• understand the epidemiology of pneumonia, the clinical and radiological criteria for diagnosis, and the relevant “typical” and “atypical” pathogens causing community-acquired and hospital-acquired pneumonia, including tuberculosis, as well as the prognostic implications.
• be able to differentiate pneumonia and tuberculosis pathogens based on the pathological tissue changes caused by these pathogens.
• understand the epidemiology of viral hepatitis, the clinical criteria for diagnosis, potential complications, and the association with malignant and other diseases.
• be familiar with the laboratory diagnostics of viral hepatitis (including herpes viridae) depending on the stage of infection and the clinical presentation.
• understand the relevant anti-infective agents and be able to evaluate their appropriate use in the prophylaxis and treatment of infectious diseases, taking into account the principles of rational antibiotic therapy to prevent antimicrobial resistance.

Digital assessments were administered on tablets using the tExam application. Digital scoring was conducted with the EX3 software. Both programs were provided by the Umbrella Consortium for Assessment Networks (UCAN; Heidelberg, Germany), and operated by the Institute for Communication and Assessment Research, a non-profit organization (Heidelberg, Germany).

A total of 350 students participated as teams. Team formation process was carried out following a transparent protocol as previously suggested by other studies on team-based learning [18]. Examinees were admitted to the examination room as pairs only with their seatmate from the laboratory course serving as partner. Only in very few cases without a seatmate, students were randomly assigned a partner immediately prior to the exam. During the examination, low-voice discussion with the partner was permitted, in order to agree on a joint final response required for each item. The examination time was limited to 60 min (according to 3 min per KFQ). The time allocation per question was determined by doubling the allotted 90 s to answer a stand-alone single-choice question in the national licensing examinations to ensure that students also had sufficient opportunity for discussion.

### Survey

Immediately after the exam, students were invited to complete a questionnaire. No incentives were offered for participation.

### Questionnaire development

The post-examination questionnaire was developed specifically for this study, as no validated instrument was available to capture student perceptions of collaborative summative examinations in medical education (see supplementary information). Questionnaire development followed an iterative, expert-driven process. First, an expert group consisting of faculty members with experience in medical education, assessment design, and curriculum development defined eight relevant domains based on group discussion and consensus: (i) perceived stress compared to individual examinations, (ii) preparation effort, (iii) enjoyment of collaboration, (iv) quality of exchange, (v) perceived benefit from the partner’s input, (vi) perceived benefit from one’s own contributions, (vii) consensus-building in decision-making, and (viii) perceived examination success. Based on these domains, individual questionnaire items were formulated and refined through several rounds of discussion within the expert group to ensure content relevance and clarity. Following initial development, the questionnaire was reviewed and tested by additional team members who had not been involved in the item generation process. Their feedback focused on item comprehensibility, wording, and perceived ambiguity, and resulted in minor revisions to improve clarity and face validity.

**Fig. 1:**
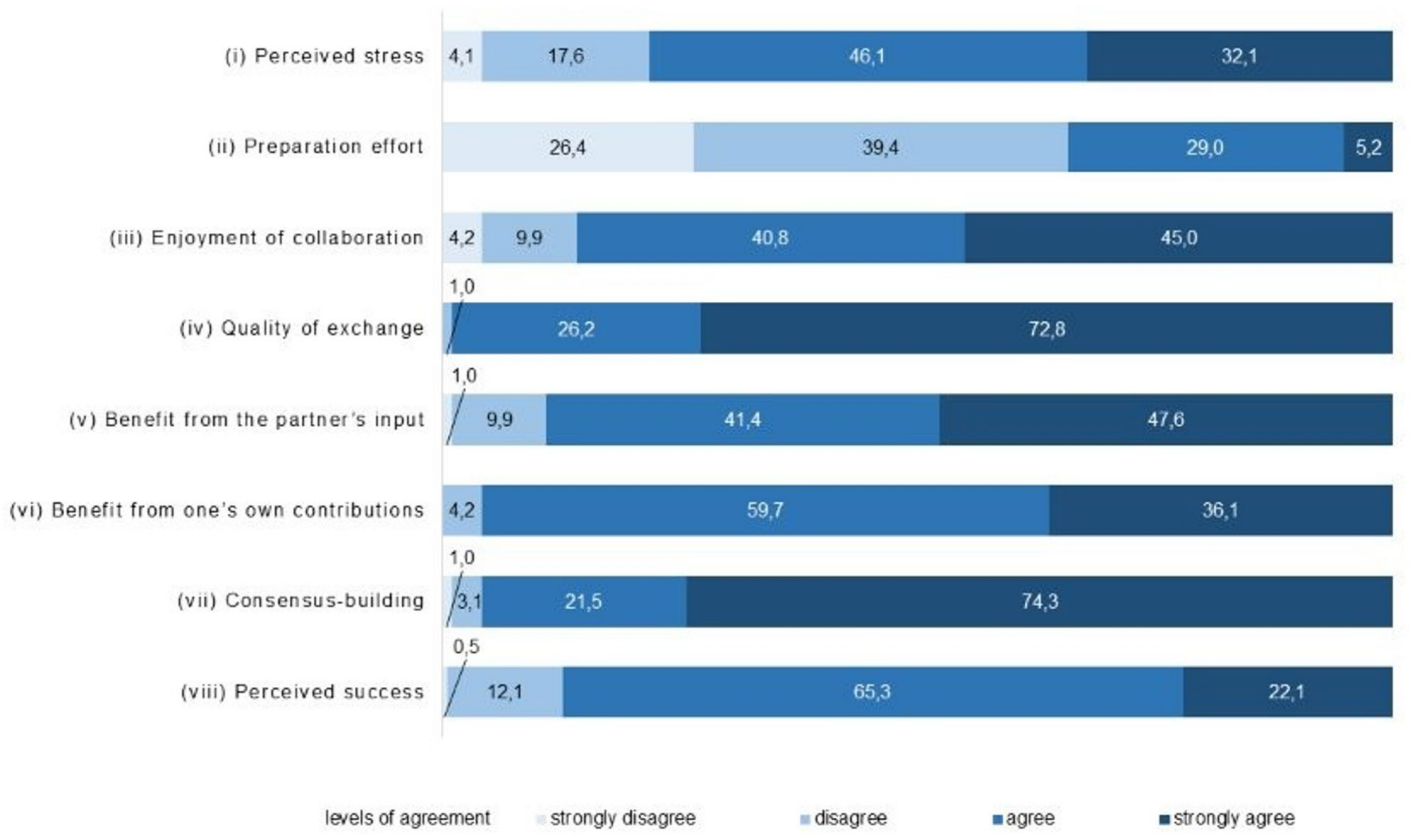
Distribution of survey responses [%] expressed as level of agreement with the following specified items: (i) “I experienced considerably less anxiety before this examination compared to prior individual written exams”, (ii) “the examination format resulted in a somewhat lower preparation effort for this exam compared to an individual examination”, (iii) “collaboratively working on the solutions was enjoyable”, (iv) “the interaction with my partner was constructive”, (v) “I benefited from my partner’s knowledge”, (vi) “my contributions were essential to solving the tasks”, (vii) “decisions regarding the selected answers were made by consensus” and (viii) “from my perspective, we performed well in the examination”

Responses were recorded using a four-point Likert scale (“strongly agree,” “agree,” “disagree,” “strongly disagree”) to avoid a neutral midpoint and to encourage respondents to indicate a clear tendency [19]. The exact wording of all items is provided in Fig. 1. In addition to the structured items, optional free-text fields were included to allow students to elaborate on their experiences and to capture aspects not covered by the predefined domains.

The survey was designed using SoSci Survey (SoSci Survey GmbH, Munich, Germany) [20] and carried out online via quick response (QR) code immediately after the exam. Participation was voluntary and conducted on students’ personal mobile devices. Entry of the tablet identification number enabled subsequent linkage of questionnaire responses to examination performance.

Survey data were exported in CSV format and analyzed with Microsoft Excel (Microsoft Office Professional Plus 2016). Results were presented descriptively and illustrated graphically. Free-text responses were categorized thematically. For all tablet numbers provided, overall examination performance was compared with students’ self-assessed success.

## Results

### Pass rate

A total of 350 students (175 pairs) participated in the examination with a mean examination score of 14.6 out of 20 points (73%). Of the 350 participants, 326 students (163 pairs) passed with at least 60% of the maximum achievable score, resulting in an overall pass rate of 93.1%. According to the study regulations of Goethe University Frankfurt, the sliding-scale clause was calculated, but not applied (lowering of the passing threshold such that all candidates whose score falls no more than 22% below the average score of all examinees are deemed to have passed the examination) demonstrating that the difficulty level of this assessment was appropriate.

### Survey results

Results of the survey are displayed in Fig. 1. A total of *n* = 193 students (193/350, 55.1%) initiated the voluntary survey, however, only *n* = 190 (190/350, 54.3%) completed it in full.

Most respondents reported lower (*n* = 89/193, 46.1%) or substantially lower (*n* = 62/193, 32.1%) levels of pre-exam anxiety compared to traditional individual written examinations. The majority of participants disagreed (*n* = 76/193, 39.4%) or even strongly disagreed (*n* = 51/193, 26.4%) that their preparation effort was lower than that required for individual examinations. Collaborative problem-solving was described as enjoyable (*n* = 78/191, 40.8%) or very enjoyable (*n* = 86/191, 45.0%). Nearly all respondents (99%) perceived teamwork during the exam as constructive (*n* = 50/191, 26.2%) to highly constructive (*n* = 139/191; 72.8%).

Most students reported a substantial (*n* = 79/191, 41.4%) or very substantial (*n* = 91/191, 47.6%) benefit from their partner’s knowledge. Nearly all respondents also perceived their own contribution as essential (*n* = 114/191, 59.7%) or very essential (*n* = 69/191, 36.1%) to the examination outcome. With (*n* = 41/191, 21.5%) agreement or (*n* = 142/191, 74.3%) strong agreement, consensus-based decision-making was reported by the majority of participants.

### Self-assessment

With regard to perceived performance, most respondents were confident (*n* = 124/190, 65.3%) or even very confident (*n* = 42/190, 22.1%) about having performed well in the examination (Fig. 1). However, the overall pass rate was 93.1% (*n* = 326/350) and the pass rate among the students who participated in the survey was even higher (*n* = 184/190, 96.8%), both clearly exceeding the proportion of students who reported confidence in their performance.

In total, 91.7% (*n* = 177/193) of students who participated in the survey, but only 25% (*n* = 6/24) of the students who failed the examination supplied their tablet number in the survey. Thus, conclusions regarding miscalibration of self-assessment remain exploratory. Among the *n* = 16/350 (4.6%) top performers with 18 or 19 correct answers, *n* = 7/16 (43.8%) completed the survey and predominantly rated their performance positively (*n* = 6/7, 85.7%), while only one (*n* = 1/7, 14.3%) strongly endorsed the statement regarding good self-assessment.

### Free-form text comments

Optional free-form text comments were provided by *n* = 80/190 students (42.1%) that completed the survey in full. Free-text comments were analyzed using descriptive thematic categorization. Initially, two summative categories were derived from the predefined questionnaire domains („*perception of case-based team assessment*“ and „*perception of fairneess*,* balance of contributions*“). During review of the responses, two additional categories were created where comments addressed aspects not covered by these domains, including technical aspects of digital assessment and organizational factors of the examination setting. The categorization was performed to provide contextual information complementing the quantitative survey results and was not intended as a formal qualitative analysis. Therefore, no formal codebook, inter-rater reliability assessment, or qualitative saturation analysis was undertaken.

#### Perceptions of case-based team assessment

Positive remarks included favorable perceptions of the case-based assessment format, which was described as helpful and conductive to learning, as well as appreciation of the collaborative exam format, which was perceived as reducing pressure.

- *“Overall a good experience. I liked the well-constructed cases and the requirement to lock in answers before moving on to the next question.”*
- *“Great format! Understanding an entire patient presentation is enjoyable and a great approach. I wish more exams were structured this way […]”*.
- *“This type of exam felt very relaxed*,* and we learned the material much better with the help of the cases.”*
- *“A great opportunity to work on solutions together*,* reduces pressure.”*
- *“I think the whispering exam is a fantastic way to review the entire practical course; without it*,* I probably would not have done so.”*
- *“Much better than individual exams.”*

Critical comments regarding the case-based team assessment were also expressed:

- *“[…] The cases were long and took a long time to read in the first place.”*
- *“[…] It can become difficult when you disagree (with your partner) […]”*.
- *“[…] I do not find this type of exam pleasant and hope not to experience similar situations again.”*
- *“[…] The argument that this serves as preparation for the national licensing examination seems premature in the 5th semester.”*

#### Perceptions of fairness and balance of contributions

Reported criticisms included concerns about disadvantages when paired with a weaker partner.

- *“[…] I do not think it is fair because it does not test individual knowledge*,* and someone may fail because their partner insisted on an incorrect answer…”*.
- *“[…] If someone has a strong partner*,* they might pass even though they would not have managed on their own.”*
- *“[….] I was stressed because my work pace was slower than my partner’s.”*

#### Transition to digital assessment

Issues related to digital assessment mainly included technical aspects such as poor display of vignettes or formatting problems.

- *“I generally find exams on the iPad less practical. Some things were confusing […]*.
- *“At one point*,* only four questions were displayed*,* the participant’s name was not shown*,* and the image appeared upside down. […]”*.
- *“I would prefer the exam to be paper-based.”*

#### Perception of work atmosphere and organizational aspects

Several students described the exam atmosphere as disruptive, particularly due to low-voice discussions and disturbances caused by peers leaving the room before the exam ended.

- *“I found it extremely difficult to concentrate on the cases and questions with all the background noise […]”*.
- *“[…] Concentration is already difficult with the whispering*,* but once people start packing up*,* it becomes really exhausting.”*

## Discussion

This study evaluated the feasibility and student perceptions of a collaborative, case-based summative examination in undergraduate medical education. Despite the only moderately high participation rate, strong examination performance and generally positive student feedback support the practical feasibility of implementing such an assessment format within a formal curriculum. Thus, the efforts for implementation of this sustainable, case-based teaching concept including clearly defined learning objectives and the final case-based assessment for the laboratory course in hygiene, microbiology and virology for third year medical students at the University hospital in Frankfurt [16] can be rated as positive. The curricular learning objectives, teaching methods and assessment format are in accordance with the principles of constructive alignment. This includes that instructors are expected to clearly communicate learning objectives, align teaching methods accordingly, and ensure that assessments validly reflect students’ achievement of these objectives.

The understanding of underlying concepts relevant to decision-making processes is particularly important in the context of the increasing complexity involved in managing infectious patients in the field of hygiene, microbiology and virology. Research in medical education has demonstrated that the stepwise clinical reasoning required to solve clinical cases represents a particularly valuable competency for future physicians [17]. The shift from a case-based paper examination to a digital assessment format at the end of the practical course in hygiene, microbiology, and virology now urges students to apply previously acquired knowledge to similar clinical cases addressed during the preceding laboratory sessions and to make key diagnostic and therapeutic decisions by the use of KFQ.

KFQ are known as particularly suitable, as they target critical decisive moments in clinical reasoning more effectively than conventional multiple-choice questions. To fully exploit their potential, a digital examination format is advantageous, allowing progressive disclosure of case information [17]. In the present survey, the numerous positive free-text comments on this authentic case-based approach simulating realistic, complex clinical decision-making situations clearly demonstrate its acceptance among students. These findings are consistent with those of Grumer et al., who showed that KFQ reliably demand clinical reasoning and are considered being relevant and supportive of learning [21]. Moreover, these data are in agreement with the findings of Huwendiek et al., who found that KFQ, compared to context-rich single-best-answer questions, are perceived as more realistic, challenging, and motivating, thereby fostering engagement with clinical decision-making [22].

The concept of team-based learning includes individual work, teamwork and immediate feedback through individual and team readiness assurance tests, and has already been successfully implemented in more than sixty U.S. and international health science professional schools [18]. However, to our knowledge, the Frankfurt whisper exam was the first collaborative exam officially implemented into a German medical school curriculum to diversify the assessment portfolio. This approach is in agreement with prior studies by Holzinger et al. that suggested a students’ general preference for a mixture of assessment formats. While multiple-choice questions were valued in this study for their objectivity and targeted preparation, students favored a combination of short-answer with oral formats to foster long-term knowledge retention [23]. Data on the perception of team-based assessments by medical students, however, remain limited. The regulations of the Frankfurt ‘whisper exam’ (approved by the medical faculty) that only allow participation in pairs and assess passing or failing as a team, were designed to force participants to apply collaborative skills as part of their medical competencies to be acquired during undergraduate training. The very positive overall perception of this team-based format by the majority of participants in this study may, at least in part, result from another key finding of this survey, which revealed that students experienced less stress before this team examination than before a comparable individual exam. Important factors associated with reduced stress levels for collaborative exams previously reported [11] included mitigating negative social comparisons (as expressed in the proverb “a problem shared is a problem halved”) or individual test anxiety [24], as well as distributing decision-making responsibility among multiple members and collectively managing uncertainties [25].

The fact that most students in this survey reported a comparable level of preparation to that for an individual examination despite the announced team-based “passing or failing as a team” regulations clearly shows that the exam format did not negatively affect their preparation efforts. On the contrary, it demonstrates a significant awareness of individual responsibility within the team. Similar effects have been observed in studies of collaborative anatomy exams, where students reported an increased sense of responsibility and benefited from improved team performance and learning gains through discussion [26]. In the present survey, the majority of students rated their experience with teamwork in the whisper exam as “constructive” and even “enjoyable”. These data support the hypothesis of Wissman et al. that testing is more effective and “fun” when implemented in a group versus alone [13]. Moreover, these results reflect the importance of collegial exchange for productivity and a respectful working atmosphere from the students’ perspective and are in accordance with those of several previous studies demonstrating positive effects of collaboration on communication skills in biomedical examinations [27].

Regarding fairness and balance of contributions, the vast majority of students emphasized both their own input (95.8%) and their partner’s input (89.0%) as essential to success. The slight asymmetry in perception of the contribution levels, however, did not appear to affect consensus-based decision-making, which was reported by 95.8% of participants. Only very few students perceived disadvantages due to partner differences in ability or pace. Since stressful interactions within the team can jeopardize the success of any team-based examination as shown in one previous study [27], medical students need to train their competencies of communication and collaboration challenged in team examination situations early in the curriculum. Thus, in preparation for the dynamics of cooperative decision-making process in the Frankfurt ‘whisper exam´ undergraduate medical students are guided through case discussions in groups, and joint performance of practical exercises throughout the entire preceding laboratory course of the hygiene, microbiology and virology. However, the fact that professional practice requires effective collaboration with any given colleague, students need to accept the challenge of cooperation with partners of varying skills and engagement.

Students’ self-assessment showed good overall alignment with their actual performance, though with a tendency toward underestimation: Even among the top-performing students only one out of seven fully endorsed his/her success. Such underestimation may reflect uncertainty due to the novel format of the exam or phenomena such as the impostor effect [28], which is particularly common among high-achieving individuals, or the Dunning–Kruger effect, where higher-performing students tend to underestimate their abilities [29]. The latter contrasts with other studies showing overestimation of competence in practical tasks such as hand hygiene or basic life support [30, 31]. This suggests that the accuracy of self-assessment may be task-dependent and influenced by contextual factors such as routine experience and confidence in one’s abilities.

The transition to a digital assessment had already been examined in earlier faculty surveys (data not published) and was well received in the present study by the majority of students, as only a small proportion indicated a preference for paper-based examinations in their free-form text comments. These students considered taking exams on the iPad to be less practical, particularly with regard to technical problems and reading lengthy case descriptions. This is indeed surprising, given that Generation Z is generally attributed a high affinity to IT-based processes [32]. One possible explanation could be that the students wish to avoid the additional stress associated with operating tablets during the examination, perceiving this as an extra burden.

Students’ criticism regarding the perceived length of case vignettes (displayed at any corresponding KFQ) is a potentially valid learner feedback. However, studying lengthy case descriptions under time pressure is a commonly encountered and daily clinical scenario in the management of patients with infectious diseases. Moreover, this digital format prepares students for the national licensing examination after the 5th year of medical studies (prior to the final clinical year), which involves extensive case-based scenarios and is expected to move on to digital delivery (17). In this regard, our digital case-based assessment concept was proactively adapted in anticipation of this expected change.

Despite the numerous parallel conversations during the examination, the overall noise level in the room remained low, and only a small number (*n* = 6/80; 7.5%) of students indicated in their open comments of the survey that the noise impaired their ability to concentrate. However, the generally strong performance observed in this examination indicates that the majority of the cohort is capable of maintaining concentration and perform effectively even in busy environments under time pressure which represents an essential quality in future physicians.

Several free-text comments referred to minor organizational shortcomings. Technical issues such as formatting errors had already been documented in prior faculty surveys and are currently being addressed. Content-related ambiguities and unclear wording have been subjected to critical error analysis as part of ongoing quality assurance.

The key limitations of this study include the use of a non-validated questionnaire, a moderate overall response rate of 54.3%, and its single-institution, single-cohort design. Therefore, the novel study format should ideally be tested in a multicenter approach, which would necessitate the resolution of several challenging issues beforehand, making these studies difficult to conduct: (i) whether the “pass or fail as a team” concept is legally unassailable, (ii) whether the computer technology used for examination is comparable, and (iii) whether students are adequately prepared for a team-based exam format in the preceding practical course. Additional studies might help to evaluate whether this team-based approach may also be transferable to other established examination formats such as Objective Structured Clinical Examinations (OSCE) or oral examinations.

## Conclusions

This study demonstrates that the collaborative, digital case-based “whisper exam” is a feasible and well-accepted assessment format in undergraduate medical education. Students perceived the examination as authentic, constructive, and less stressful than comparable individual assessments, while maintaining high levels of personal responsibility and preparation. The findings support the educational value of key feature questions as a competency-based assessment format for fostering clinical reasoning skills essential for future physicians. Because of the challenges it places on teamwork, this examination format possesses particular innovative potential.

## Supplementary Information

Supplementary Material 1.

Supplementary Material 2.

## Data Availability

Summarized and anonymized results (excel-sheet) will be provided upon request by the corresponding author.

## Abbreviations

“whisper exam”: case-based collaborative summative examination
KFQ: Key-feature questions
QR code: Quick response code
OSCE: Objective Structured Clinical Examinations
