# Supplementary material for: The Frankfurt ‘whisper exam’ - a case-based collaborative summative examination to assess clinical decision-making skills in hygiene, microbiology and virology– feasibility and evaluation
Source: BMC Med Educ. 2026 Aug 1;26:1229. doi: 10.1186/s12909-026-10018-y (PMC13428416; doi:10.1186/s12909-026-10018-y)
Supplement: Supplementary file 1 — Supplementary Material 1. [file 12909_2026_10018_MOESM1_ESM.docx]

**CROSS Checklist**

**CROSS Item Requirement Compliance/Comments**

Title/Abstract Identify survey Survey is described in the abstract

Structured Abstract Completed

Introduction Background and rationale Explained

Objectives Stated

Methods Study design Prospective single-center descriptive

feasibility study

Setting and dates Setting is reported; exam period was

added to project description (lines 123-4)

Target population Third-year medical students

Eligibility criteria All enrolled students were eligible

Sampling strategy Census of one student cohort

Recruitment Invitation immediately after the exam

Voluntary participation Reported

Incentives No Incentives were offered,

added to project description (lines 160-1)

Questionnaire Questionnaire development Described

Theoretical basis Expert consensus; no validated instrument available

Validity evidence Face validity reported; no formal psychometric validation was performed

Pilot/pretest Internal review reported; no formal pilot study was conducted

Questionnaire availability Provided in supplementary files

Number of items Number of eight Likert items mentioned

Response scale Four-point Likert scale

Open-ended questions Free text option reported

Survey Administration mode Online via QR code

Survey platform SoSci Survey

Timing Immediately after exam

Completion time Not assessed, estimated 3-5 minutes

Reminder strategy Not applicable

Duplicate prevention Entry of tablet ID prevented more than two submissions

Ethics Ethic approval Not required

Informed consent Not required

Confidentiality Tablet IDs were used solely for linkage and handled confidentially

Data analysis Statistical analysis Descriptive statistics reported

Software Excel reported

Missing data Incomplete questionnaires were evaluated to the point of completion

Qualitative analysis Descriptive thematic categorization explained

Results Number of students invited 350

Response rate 54,3%

Completion rate Reported

Participant characteristics Demographics of cohort not available;

not relevant

Discussion Interpretation Completed

Limitations Non-validated questionnaire, moderate response rate, single-institution, single-cohort design addressed

Generalizability Transferability discussed
