# Supplementary material for: The Frankfurt ‘whisper exam’ - a case-based collaborative summative examination to assess clinical decision-making skills in hygiene, microbiology and virology– feasibility and evaluation
Source: BMC Med Educ. 2026 Aug 1;26:1229. doi: 10.1186/s12909-026-10018-y (PMC13428416; doi:10.1186/s12909-026-10018-y)
Supplement: Supplementary file 2 — Supplementary Material 2. [file 12909_2026_10018_MOESM2_ESM.docx]

**Questionnaire development**

The post-examination questionnaire was developed specifically for this study to capture student perceptions of collaborative summative examinations in medical education, as no validated instrument was available to capture student perceptions of collaborative summative examinations in medical education. Questionnaire development followed an iterative, expert-driven process. First, an expert group consisting of faculty members with experience in medical education, assessment design, and curriculum development defined eight relevant domains based on group discussion and consensus: (i) perceived stress compared to individual examinations, (ii) preparation effort, (iii) enjoyment of collaboration, (iv) quality of exchange, (v) perceived benefit from the partner’s input, (vi) perceived benefit from one’s own contributions, (vii) consensus-building in decision-making, and (viii) perceived examination success. Based on these domains, individual questionnaire items were formulated and refined through several rounds of discussion within the expert group to ensure content relevance and clarity. Following initial development, the questionnaire was reviewed and tested by additional team members who had not been involved in the item generation process. Their feedback focused on item comprehensibility, wording, and perceived ambiguity, and resulted in minor revisions to improve clarity and face validity. Responses were recorded using a four-point Likert scale (“strongly agree,” “agree,” “disagree,” “strongly disagree”) to avoid a neutral midpoint and to encourage respondents to indicate a clear tendency. The exact wording of all items is provided below. In addition to the structured items, optional free-text fields were included to allow students to elaborate on their experiences and to capture aspects not covered by the predefined domains.

**Anleitung zum Fragebogen**

**Direkt nach Abgabe der Klausur möchten wir Sie bitten, an unserer Umfrage zur Murmelprüfung teilzunehmen. Nur durch Ihre Rückmeldung ist ein konstruktiver Verbesserungsprozess möglich. Für die Umfrage benutzen Sie bitte Ihr mobiles Endgerät und loggen sich mit dem im Hörsaal angezeigten QR-Code ein. Für Ihre Zeit und Mühe bedanken wir uns schon im Voraus bei Ihnen sehr herzlich.**


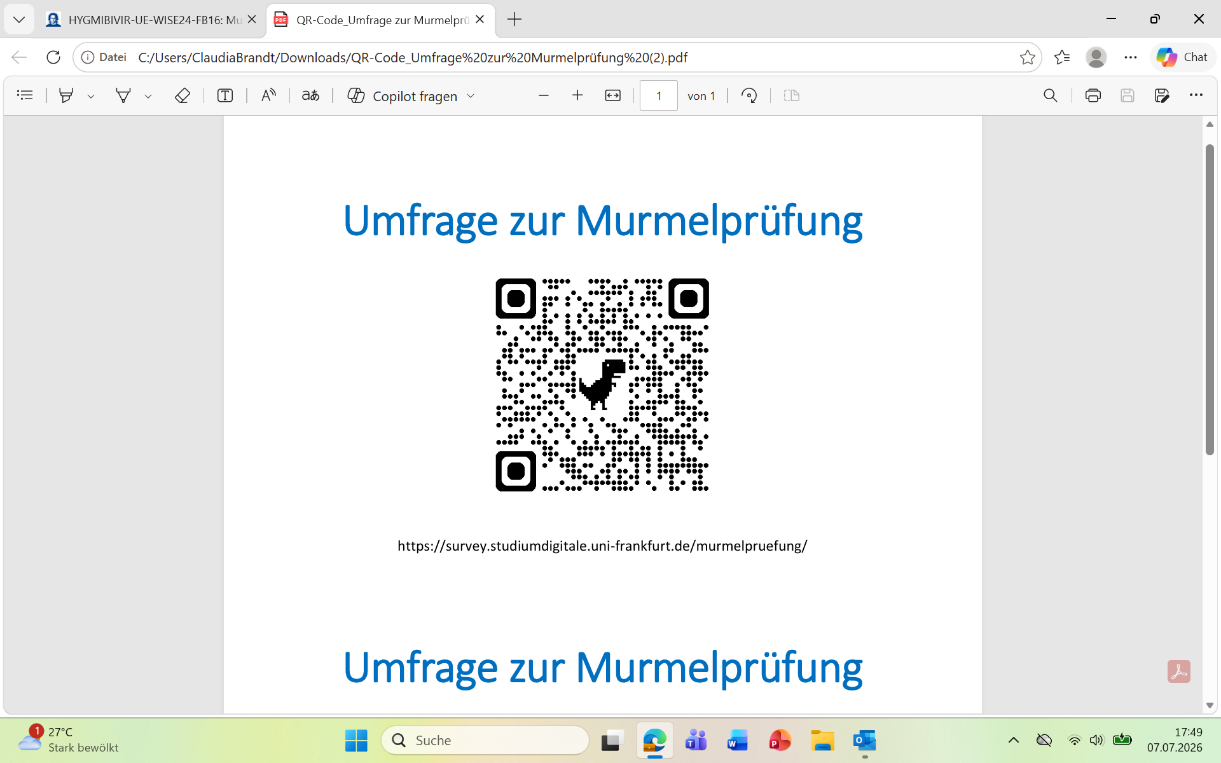


**Fragebogen**

1. **Prüfungsvorbereitung:** Meine Anspannung vor dieser Prüfung war deutlich weniger ausgeprägt als vor bisherigen schriftlichen Einzelprüfungen.
2. **Prüfungsvorbereitung:** Prüfungsvorbereitung: Aufgrund des Prüfungsformats war mein Vorbereitungsaufwand für diese Klausur eher geringer als für eine Einzelprüfung.
3. **Teamarbeit:** Das gemeinsame Erarbeiten der Lösungen hat mir Spaß gemacht.
4. **Teamarbeit:** Der Austausch mit meinem Partner/ meiner Partnerin war konstruktiv.
5. **Teamarbeit:** Ich habe stark von dem Wissen meines Partners/ meiner Partnerin profitiert.
6. **Teamarbeit:** Meine Beiträge haben wesentlich zum Lösen der Aufgaben beigetragen.
7. **Teamarbeit:** Die Entscheidungen für die gewählten Antworten haben wir einvernehmlich getroffen.
8. **Leistungseinschätzung:** Nach meiner Einschätzung ist uns die Prüfung gut gelungen.

**Questionnaire guide**

**Immediately after submitting your exam, we kindly ask you to participate in our survey on the Whisper examination. Your feedback is essential for supporting a constructive process of continuous improvement. To complete the survey, please use your mobile device and access it by scanning the QR code displayed in the lecture hall. We greatly appreciate your time and effort and thank you in advance.**


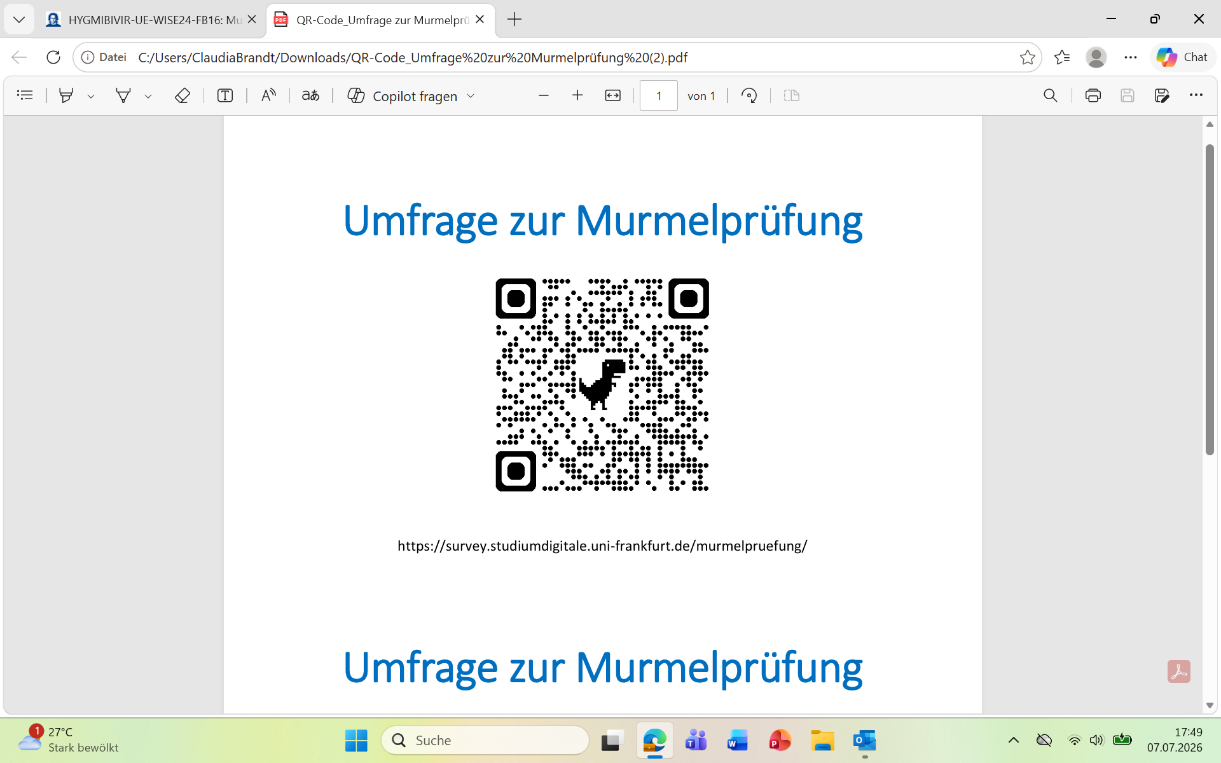


**Questionnaire**

1. **Exam preparation:** My level of anxiety prior to this exam was lower than in previous written individual exams.
2. **Exam preparation:** Due to the exam format, the amount of preparation required for this exam was lower than for an individual exam.
3. **Teamwork:** I enjoyed the collaborative development of the solutions.
4. **Teamwork:** The interaction with my partner was constructive.
5. **Teamwork:** I benefited from my partner’s knowledge.
6. **Teamwork:** My contributions contributed significantly to solving the tasks.
7. **Teamwork:** The decisions regarding the selected answers were made jointly.
8. **Performance assessment:** In my assessment, the exam was completed successfully.
